# “Old is gold”

**DOI:** 10.4103/0972-0707.48832

**Published:** 2008

**Authors:** AP Tikku

**Affiliations:** Professor, Department of Conservative Dentistry and Endodontics, and Dean, Student Welfare, C.S.M. Medical University, Lucknow, India

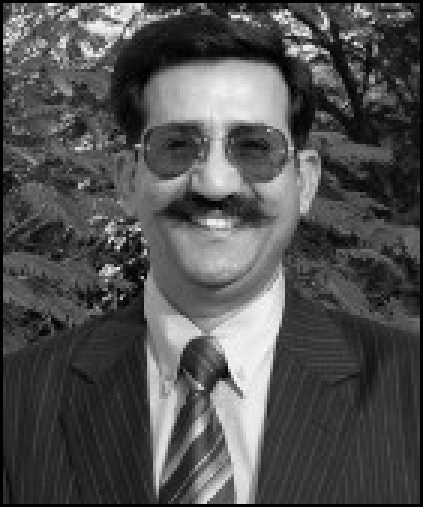



Materials like gold and silver amalgam have been used for a hundred years with great benefit and advantage, as restoration, in mankind. Although material science has evolved to tremendous levels in the last hundred years, backed by innumerable publications all over the world, it is indeed remarkable that among the numerous restorative materials introduced to date, clinically, none have really been able to better the qualities of gold and silver, as restorations, in terms of longevity, durability, and compatibility. I have, in my own mouth, silver restorations that are more than 20 years old and still doing fine.

It is also a matter of concern that many more of us have started giving preference to materials other than gold and silver, for restorative purposes. There is a definite decline in the use of gold inlays and class 11 silver restorations in the recent past. Our specialty has taken tremendous strides in the past two decades and is today at the top in dentistry. To take it further we must emphasize on permanent treatment for our patients, rather than go for what I would call ad-hoc or add-on or cut-and-paste dentistry.

Frankly speaking, another matter that concerns me is that some of the top office bearers of our federation have become speakers in the student convention / national conferences. In my opinion the office bearers should refrain from speaking at national conferences during the term they hold their posts. It will augur well for the association.

I am really impressed by the quality and content of our national journal and my congratulations to the efforts put in by the editor and his team of hard working colleagues. My best wishes to all.

